# Two conformational forms of target-bound iC3b that distinctively bind complement receptors 1 and 2 and two specific monoclonal antibodies

**DOI:** 10.3109/03009734.2010.528465

**Published:** 2011-02-11

**Authors:** Ulf R. Nilsson, Lillemor Funke, Bo Nilsson, Kristina N. Ekdahl

**Affiliations:** ^1^Division of Clinical Immunology, Rudbeck Laboratory C5, Uppsala UniversitySweden; ^2^Department of Natural Sciences, Linneaus University, KalmarSweden

**Keywords:** Complement C3, complement receptors, immunoglobulin G (IgG), monoclonal antibodies, neo-epitopes

## Abstract

**Introduction:**

The complement system is an essential part of the immune system of vertebrates. The central event of the complement activation cascade is the sequential proteolytic activation of C3, which is associated with profound alterations in the molecule's structure and conformation and is responsible for triggering most of the biological effects of complement.

**Material and methods:**

Here, we have studied the conformation of C3 fragments deposited onto an IgG-coated surface from human serum during complement activation, using a set of unique monoclonal antibodies (mAbs) that are all specific for the C3dg portion of bound iC3b.

**Results:**

We were able to identify two conformational forms of target-bound iC3b: the first recognized by mAb 7D18.1, and the second by mAb 7D323.1. The first species of iC3b bound recombinant complement receptor 1 (CR1), while the second bound CR2. Since CR1 and CR2 are expressed by different subsets of leukocytes, this difference in receptor-binding capacity implies that there is a biological difference between the two forms of surface-bound iC3b.

**Conclusion:**

We propose that mAbs 7D18.1 and 7D323.1 can act as surrogate markers for CR1 and CR2, respectively, and that they may be useful tools for studying the immune complexes that are generated in various autoimmune diseases.

## Introduction

The complement system is an essential part of the innate immune system with the capacity to discriminate between self and non-self. It is also an important player in pathological processes, since it also recognizes altered self in clinical settings such as reperfusion injury and transplantation. The complement system is a removal system, in that it eliminates micro-organisms (bacteria, viruses, parasites), immune complexes, and apoptotic cells from the body. Complement factor C3 is the key component in this system, and its cleavage to C3b by labile composite enzymes (convertases) elicits inflammation and cell lysis via target-bound C3 fragments (the anaphylatoxins C3a and C5a) and the generation of the membrane attack complex, C5b-9.

**Figure 1.**
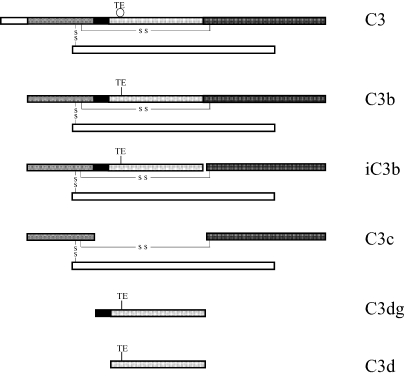
Sequential digestion of C3, generating C3b, iC3b, C3c, C3dg, and C3d. Activation of C3 leads to disruption of the thiol ester (TE circular), which then establishes covalent bonds (TE linear). The interchain disulfide bonds are indicated (S S).

During its cleavage to C3b and iC3b, the C3 molecule undergoes several profound structural and conformational changes. These alterations in structure and conformation have recently been elucidated in a series of reports on the 3-D crystal structure of the molecule (1–5). After cleavage of C3 by the convertases, the so-called thiol ester domain (TED) is totally dislocated and moved into a position that allows covalent binding of the molecule to a target surface. Digestion of C3b to iC3b by factor I affects the molecule further by releasing the C3f fragment and displacing the C3c domain from the TED. Finally, factor I further cleaves iC3b, generating the fragments C3dg (TED) and fluid-phase C3c (Figure 1).

The conformational changes that occur during the activation of C3 are reflected in alterations in the exposure of neo-epitopes on the surface of the C3 molecule. Immunization of rabbits and mice with the native molecule as antigen tends to produce antibodies directed toward both the fluid-phase and the target-bound molecules. In an attempt to generate monoclonal antibodies (mAbs) that specifically recognize the bound conformations of C3 fragments, we have produced mAbs against reduced polypeptide chains of C3. A number of mAbs directed against surface-bound C3 fragments (C3b, iC3b, C3dg) have been generated (6), some of whose epitopes in the C3 molecule have been mapped using cDNA-expressed and synthesized peptides (7,8). Several of these antibodies were directed against the N terminus of the C3dg fragment and recognized the iC3b and C3dg fragments (9). All of these mAbs, bound to epitopes that are not totally exposed in the 3-D crystal structure, indicate that there are still unidentified conformational states that are associated with binding of the molecule to target surfaces (1).

Binding of C3b to a target surface is associated with exposure of epitopes in C3 that act as ligands for complement receptors 1–4 (CR1–4). Two of these receptors, CR1 (CD35) and CR2 (CD21), are short consensus repeats (SCR) consisting of 30 (10,11) and 15 or 16 (due to alternative splicing) (12,13) residues, respectively. CR1 is expressed on erythrocytes and various phagocytes, whereas CR2 is specific for lymphocytes and follicular dendritic cells. In particular, B-cells express a large number of CR2 molecules.

In the present paper, we have further characterized the reactivity of four C3dg mAbs, 7D18.1, 7D84.1, 7D264.6, and 7D323.1, with regard to their binding to different conformational states of surface-bound C3b/iC3b that are associated with the binding of complement receptors CR1 and CR2. A distinctive association was found between the binding of the mAbs and the receptors.

## Material and methods

### Serum preparations

Fresh, normal human blood was obtained from the Blood Center of the University Hospital, Uppsala. Normal human serum (NHS) was separated from blood by centrifugation at 4000 *g* for 20 min at +4°C after clotting for 2–3 h at room temperature. All analyses of complement in serum were performed on stored, frozen material after rapid thawing at 37°C. Freezing/refreezing and storage of material were done at −70°C. For some experiments, aliquots of serum were heat-inactivated by incubation for 30 min at 56°C.

### Sources and preparation of purified proteins

Human IgG (gamma globulin) was obtained from Pharmacia-Upjohn AB (Uppsala, Sweden), and bovine serum albumin (BSA) (fraction V, RIA grade) was purchased from the United States Biochemical Corporation (Cleveland, OH, USA). Soluble complement receptor 1 (sCR1) BRL 55730 was a kind gift of Dr Henry Marsh (SmithKline Beecham Pharmaceuticals, King of Prussia, PA, USA). Culture supernatants from Raji cells (Burkitt's lymphoma), kindly provided by Dr Sara Mangsbo, Rudbeck Laboratory, were used as a source for CR2.

C3 and factor I were prepared from plasma according to Hammer et al. (14) and Fearon (15), respectively. Factor H was prepared from human serum essentially according to Hammer et al., except that the first step consisted of a euglobulin precipitation (16). C3b was produced by incubating C3 with trypsin, followed by gel filtration to remove C3a, and iC3b was prepared by incubating C3b with factor I, using factor H as co-factor.

### Antibody preparations

Polyclonal (pAb) horseradish peroxidase-(HRP)-conjugated anti-human C3c and C3d antibodies, HRP-conjugated anti-mouse-Ig and non-conjugated anti-BSA pAb, and mouse monoclonal anti-human CR1 and anti-human CR2 antibodies were purchased from Dako (Glostrup, Denmark).

Mouse anti-human C3 monoclonal antibodies (mAbs) 7D18.1, 7D84.1, 7D264.6, and 7D323.1, specific for epitopes in C3dg, were produced and characterized as described previously (6,8).

### Complement activation studies

#### Microtiter plates

Complement was activated in the wells of 96-well polystyrene microtiter plates (Maxisorp) (NUNC, Roskilde, Denmark) that had been coated with IgG as described below. The conformation of the deposited C3 fragments was visualized by ELISA using polyclonal (pAb) and monoclonal (mAb) anti-C3 antibodies. Each experiment was repeated five to ten times with similar results. Furthermore, analysis of the deposited C3 fragments was performed using Western blot analysis.

#### Diluents for functional studies and ELISAs

Reagents that were tested functionally were diluted in veronal-buffered saline (VBS) consisting of 5 mM veronal, pH 7.5, with NaCl (145 mM), Ca^2+^ (0.15 mM), and Mg^2+^ (0.5 mM). Some experiments were performed in VBS supplemented with 0.1% (w/v) gelatin (GVB). Phosphate-buffered saline (PBS) containing 0.05% (v/v) Tween 20 and 0.02% (v/v) Antifoam (Dispensor-Aspirator, Pharmacia-Upjohn, Uppsala, Sweden) was used as the washing fluid. Antibody dilutions were carried out in washing fluid containing 0.1% (w/v) gelatin. Undesired protein adsorption to polystyrene plates was prevented by incubating the microtiter wells with 1% (w/v) gelatin in PBS at room temperature for 30 min.

#### Complement-activating target surfaces

Plates were incubated for 1 h at 37°C with 0.2 mL PBS/well of monomeric human IgG at 80 μg/mL, washed 3× with PBS, blocked 30 min at room temperature with 0.3 mL 1% gelatin, and rinsed with VBS. The IgG-coated plates were used immediately or after storage at −70°C, with 0.3 mL VBS being added per well.

#### Complement activation

Human serum was diluted in VBS from 100% in 3-fold steps and incubated in pre-warmed IgG-coated microtiter wells for 2.5 to 120 min, at 37°C as described elsewhere (17). The reaction was stopped by washing with washing fluid containing 10 mM EDTA. The bound C3 fragments were detected using rabbit pAbs anti-C3c and anti-C3d, as well as a panel of four mAbs against epitopes in the C3dg region of C3. The bound primary antibodies were detected using HRP-conjugated anti-rabbit immunoglobulins (pAbs) or HRP-conjugated anti-mouse immunoglobulins (mAbs).

#### Binding of complement receptors (CR) 1 and 2 to deposited C3 fragments

The ability of the deposited C3 fragments to act as ligands for CR1 and CR2 was investigated by adding recombinant sCR1 or Raji supernatants containing soluble CR2 into microtiter wells in which complement activation had been achieved as described above. After incubation at 37°C for 30 min, mAbs recognizing CR1 or CR2 were used for detection, in conjunction with HRP-conjugated anti-mouse immunoglobulins.

#### Factor I-mediated cleavage of bound C3 fragments with CR1 as co-factor

In order to promote the digestion to iC3b or C3dg of the C3 fragments deposited by complement activation in the IgG-coated microtiter wells, separate experiments were performed in which complement activation was performed as described above. Thereafter, sCR1 and heat-inactivated human serum (or buffer as negative control) were added and incubated at 37°C for 30 min to enable the factor I in serum to digest the deposited C3b, with sCR1 as co-factor. Subsequently, Raji supernatants containing soluble CR2 were added to some of the microtiter wells. Finally, bound C3 fragments and CR1 and CR2 were detected as described above.

### Western blot analysis of bound C3 fragments

Human serum (undiluted) was incubated in IgG-coated microtiter wells as described above for up to 60 min. After washing, 25 μL 0.1 M methylamine in 0.1 M glycine-NaOH buffer, pH 8, was added to each well and incubated at room temperature with agitation for 10 min. Thereafter, 25 μL SDS-electrophoresis buffer (12 mM Tris, 0.4% SDS, pH 6.8) was added to each well of the microtiter plate, which was then heated to 85°C for 5 min. After the incubation, the supernatants were loaded onto 10% SDS polyacryle amide gel electrophoresis (SDS-PAGE) gels. After electrophoretic separation, the proteins were transferred to a PVDF Immun-Blot membrane (BioRad, Hercules, CA, USA), and the membrane was blocked in 1% bovine serum albumin diluted in PBS-Tween. Proteins were detected with biotinylated pAb anti-C3d and anti-C3c, or mAb 7D264.6, followed by streptavidin-HRP and staining with 3,3′-diaminobenzidine tetrahydrochloride (Sigma, Steinheim, Germany) dissolved in PBS (1 mg/mL plus 0.5 μL H_2_O_2_/mL). Purified C3, C3b, and iC3b were used as controls.

## Results

### Epitope expression of human C3 bound to adsorbed human IgG

**Figure 2.**
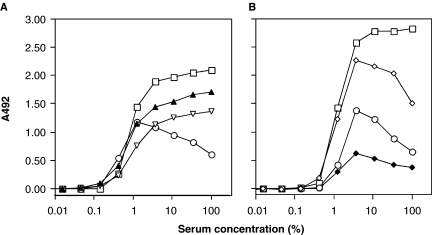
Epitope expression of human C3 bound to adsorbed human IgG. Human serum was added in 3-fold serial dilution from 100% and incubated at 37°C for 60 min (panel A) or 120 min (panel B). Bound C3 was detected using pAbs anti-C3d (filled triangles) and anti-C3c (open triangles), and mAbs 7D323.1 (squares), 7D18.1 (circles), 7D264.6 (open diamonds), and 7D84.1 (filled diamonds).

**Figure 3.**
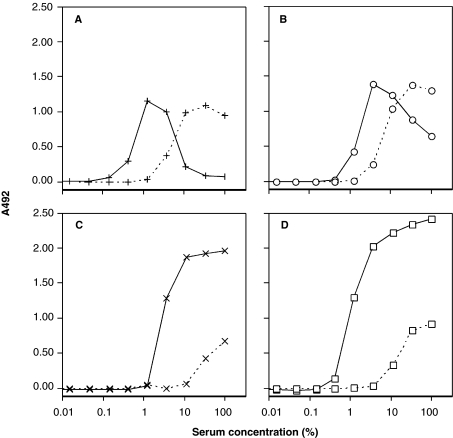
Binding of CR1 (panel A), anti-C3 mAb 7D18.1 (panel B), CR2 (panel C), and anti-C3 mAb 7D323.1 (panel D) to human C3 bound to adsorbed human IgG. Human serum (in 3-fold serial dilution from 100%) was added and incubated at 37°C for 2.5 min (dashed lines) or 60 min (solid lines).

**Figure 4.**
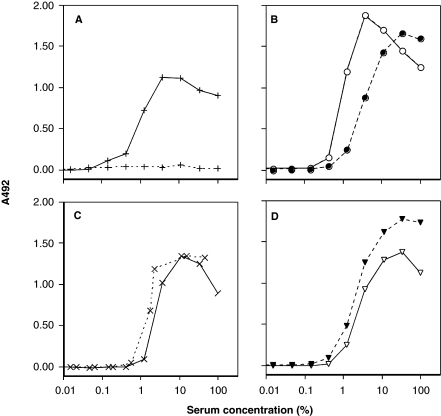
Binding of CR1 (panel A) and anti-C3 mAb 7D18.1 (panel B) and of CR2 (panel C) and anti-C3 mAb 7D323.1 (panel D) to human C3 fragments bound to adsorbed human IgG. The coated surfaces were incubated first with human serum in 3-fold serial dilution from 100% at 37°C for 60 min. After washing, heat-inactivated human serum was then added, together with buffer (solid lines) or soluble CR1 to enable digestion of deposited iC3b to C3dg (dashed lines).

Incubation of serum in wells coated with adsorbed IgG for 60 min at 37°C induced complement activation, leading to the deposition of C3 fragments on the microtiter plate surface; the highest level of binding was seen with undiluted serum, as detected by pAbs anti-C3c and anti-C3d and mAb 7D323.1 (Figure 2A). In contrast, mAb 7D18.1 showed the lowest level of binding at the highest serum concentration. The binding profiles of these two mAbs, together with those of mAbs 7D84.1 and 7D264.6, were further examined after 120 min of complement activation. Under these conditions, binding of mAb 7D323.1 was further increased, again with the highest level achieved with undiluted serum; the other mAbs showed similar binding profiles, peaking at a serum concentration of ∼3% (Figure 2B). Data presented in each panel in Figures 2–4 are from one representative experiment out of five to ten experiments which were performed with similar results.

### Receptor ligand function of human C3 bound to adsorbed human IgG

The C3 fragments deposited on adsorbed IgG as a result of complement activation of serially diluted serum were able to act as a ligand for exogenously added CR1 (Figure 3A). The CR1-binding epitopes were already exposed on C3 after 2.5 min of incubation, with maximal expression at high serum concentration (30%). After prolonged incubation (60 min), maximal binding of CR1 was seen at a lower serum concentration (3%). The binding profile of CR1 closely resembled that of mAb 7D18.1 (Figure 3B). Surface-bound C3 fragments also bound CR2, but this binding was seen only on surfaces that had been in contact with high concentrations of serum, at both 2.5 and 60 min (Figure 3C). The binding of mAb 323.1 mirrored that of CR2 (Figure 3D).

### Ligand binding of factor I-digested surface-bound C3 fragments

C3 fragments were deposited on surface-bound IgG from serially diluted serum. After removal of the serum, CR1 was added together with heat-inactivated human serum as a source of factor I to enable the digestion of the deposited C3 fragments to yield iC3b and C3dg. CR1 did not bind to the surface-bound digested C3 fragments, but binding was seen in the wells that had been incubated with CR1 and the buffer control (Figure 4A). Since there was a surplus of CR1 after addition, it was also detected at high serum concentration. After digestion, the binding profile of mAb 7D18.1 shifted from its previous maximum at 3% serum to a peak at 30% serum, suggesting a lower density of binding epitopes (Figure 4B). In contrast, factor I-mediated cleavage of the deposited C3 fragments did not affect the binding of CR2 (Figure 4C) or mAb 7D323.1 (Figure 4D).

### Western blot analysis of bound C3 fragments

**Figure 5.**
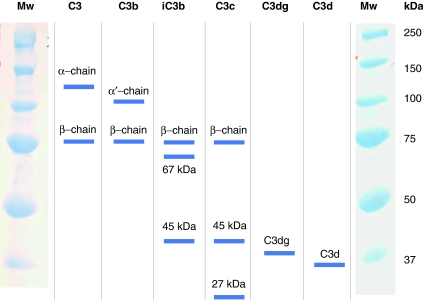
Electrophoretic pattern of polypeptide chains from the various C3 fragments. The molecular weights (Mw) of the standards are indicated.

The composition of the deposited C3 fragments was further investigated by Western blot analysis. Undiluted serum was incubated on IgG-coated ELISA plates for up to 60 min. After washing, the bound proteins were eluted with methylamine, and the eluted C3 fragments were visualized by Western blot analysis using pAbs against C3d (molecular weight ≈ 35 kDa) and mAb 7D264.6 against an epitope in C3dg, both of which also detected the corresponding epitopes in the 110-kDa α-chain of C3 and the 100-kDa α′-chain of C3b; we also used pAbs against C3c, which detected the 75-kDa β-chain and the 45-kDa polypeptide chain of C3c, including the corresponding epitopes in the 110-kDa α-chain of C3, the 100-kDa α′-chain of C3b, and the 67-kDa fragment of iC3b (Figure 5).

**Figure 6.**
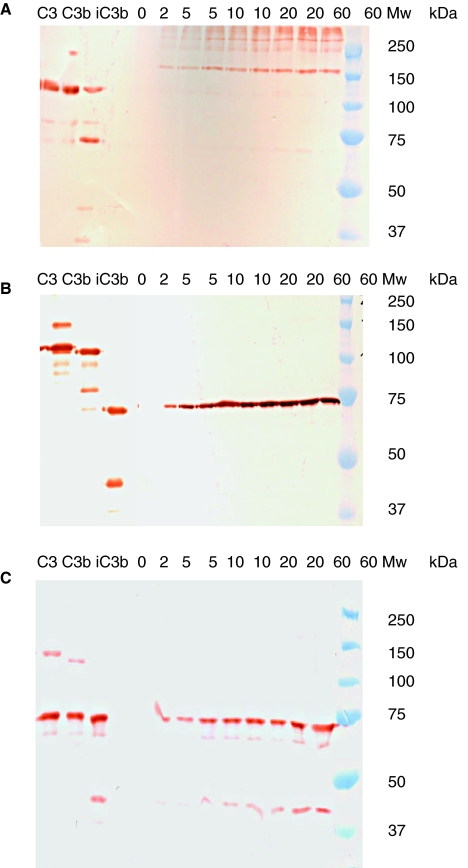
Western blot analysis of C3 fragments eluted from an IgG-coated surface exposed to undiluted human serum for 0, 5, 10, 20, or 60 min, detected using: pAb anti-C3d (panel A), mAb anti-C3 7D264.6 (panel B), and pAb anti-C3c (panel C). Reference preparations of C3, C3b, and iC3b were also analyzed, and the molecular weights (Mw) of the standards are indicated.

The staining of the anti-C3d pAbs in the controls followed the expected pattern. In the eluted samples, we saw weak staining of a band of 67 kDa (i.e. the 67-kDa band of iC3b), but no band of 40 kDa (i.e. free C3dg). However, the most abundant staining with anti-C3d pAbs was of high molecular weight material (>150 kDa) in the eluates, suggesting that most of the C3 fragments with exposed epitopes recognized by the anti-C3d pAbs were covalently bound to other proteins, producing higher molecular weight eluates. The pattern did not alter during incubation for up to 60 min (Figure 6A). Staining with mAb 7D264.6 revealed the presence of the 67-kDa fragment of iC3b at all time points, with no further digestion to C3dg over time (Figure 6B). There was no indication of heterogeneity in the size of this fragment. Staining with pAb anti-C3c confirmed the presence of iC3b by detecting the 75-kDa β-chain at all time points, as well as the 45-kDa polypeptide chain present in C3c and iC3b (Figure 6C).

## Discussion

In the present work, we have demonstrated a strict relationship between the iC3b binding patterns of 7D18.1 and 7D323.1 and complement receptors CR1 and CR2, respectively. The two antibodies were able to distinguish between two forms of iC3b, which are produced not by structural differences but apparently as a result of conformational changes in the molecule, indicating that the difference in binding of the receptors is related to a conformational change.

Monoclonal antibodies 7D18.1, 7D84.1, 7D264.6, and 7D323.1 all bound preferentially to bound C3 fragments, but the binding of 7D323.1 was less strictly limited to bound iC3b, since it also recognized the molecule in the fluid phase. The epitopes of all four antibodies are found close to each other in the N terminus of C3dg and have been pinned down to single amino acid residues (8,9,18). All four mAbs bind to C3 (929–932), but 7D264.6 also recognizes C3 (929–936), and 7D323.1 binds to rabbit C3, which has a slightly different sequence in this region: Hu929R versus Ra929N and (18) Hu932R versus Ra932Q, respectively (19,20). Thus, the location of the epitopes differs by only a few amino acid residues.

All the anti-C3dg mAbs were tested for binding to C3 fragments that had been generated and bound to IgG adsorbed in microtiter wells after incubation with NHS for 60 and 120 min, and their binding was compared with that of pAbs anti-C3c and anti-C3d, which acted as internal standards for the binding of antibodies to the bound C3 fragments. These studies showed that 7D323.1 bound to C3 fragments at both time points and at all concentrations of NHS, while 7D18.1 and the other two mAbs bound less intensely at the higher concentrations of NHS.

When the binding of mAbs was compared with that of fluid-phase recombinant CR1 (CD35) and CR2 (CD21), a close relationship was seen between the binding of the receptors and the mAbs. CR1 bound to a maximum level at a fairly low concentration of NHS, and its level of binding decreased at higher concentrations. This binding pattern was similar to that of 7D18.1, 7D84.1, and 7D264.6, as exemplified by the binding of 7D18.1. On the other hand, the binding of CR2 increased with increasing concentration of NHS. This reactivity was mirrored by the binding of 7D323.1.

There are two possible explanations for this pattern of reactivity: There may be structural differences between the two forms of iC3b, or there may be conformational changes that are associated with the two different forms of iC3b. In the transformation of C3b to iC3b, factor I cleaves the α-chain of C3b at two sites, finally releasing the C3f fragment. Theoretically, these cleavages can occur in a stepwise manner to generate two forms of iC3b, which could therefore differ in their reactivity with the receptors and mAbs. In order to rule out this possibility, we analyzed the C3 fragments by Western blotting, which revealed that the bound fragments were in the iC3b form and there were no differences associated with the various concentrations that were used, or with the length of the incubation with the NHS. No differences were found in the 45-kDa polypeptide of C3c or the C3dg fragment, which are the polypeptides which would be affected if a stepwise generation of iC3b were to occur. A further indication that the surfaces were coated with iC3b was the fact that when the bound iC3b was cleaved to generate C3dg as a result of incubation of the microtiter plates with heat-inactivated NHS and soluble CR1, the binding of CR1 to the surface was lost. This was a finding that is in agreement with the loss of affinity of the receptor for C3 fragments in the case of C3dg. The reactivity with CR2 remained, as did the binding of the mAbs, since their epitopes are present in the N-terminal portion of the C3dg molecule. Taken together, these results suggest that the differences in binding of the mAbs and complement receptors reflect conformational changes in the molecule, and not structural differences.

In summary, this study has identified two forms of iC3b that have distinctive binding reactivities with the complement receptors CR1 and CR2. These forms of iC3b were specifically detected by the mAbs 7D18.1 and 7D323.1, respectively. The reactivity of these mAbs is potentially of great interest, since the binding of iC3b to CR1 and CR2 generates totally different biological responses in the two cases. The mAbs can therefore be used as reagents to identify the different conformational and functional forms of iC3b bound to various immune complexes (21). Since immune complexes are found in many autoimmune diseases, such as systemic lupus erythematosus (SLE) and rheumatoid arthritis, assays employing these antibodies would add a new dimension to the analysis of immune complexes in patients with these types of disease.
